# Is palpable DCIS more aggressive than screen-detected DCIS?

**DOI:** 10.1016/j.sopen.2022.12.002

**Published:** 2022-12-12

**Authors:** Nina Balac, Robert M. Tungate, Young Ju Jeong, Heather MacDonald, Lily Tung, Naomi R. Schechter, Linda Larsen, Stephen F. Sener, Julie E. Lang, Kirstyn E. Brownson

**Affiliations:** a Keck School of Medicine of the University of Southern California, Los Angeles, CA 90033, USA; b Department of Internal Medicine, University of Southern California, Los Angeles, CA 90033, USA; c Department of Surgery, Catholic University of Daegu School of Medicine, Daegu 42472, Republic of Korea; d Hoag Hospital, Irvine, CA 92618, USA; e Department of Trauma Surgery and Critical Care, Vancouver General Hospital, Vancouver, British Columbia V5Z 1M9, Canada; f Department of Radiation Oncology, University of Southern California, Los Angeles, CA 90033, USA; g Department of Radiology, Division of Women's Imaging, University of Southern California, Los Angeles, CA 90033, USA; h Division of Breast, Endocrine, and Soft Tissue Surgery, Department of Surgery, Norris Comprehensive Cancer Center, University of Southern California, Los Angeles, CA 90033, USA; i Department of Surgery, LAC+USC (LA County) Medical Center, Los Angeles, CA 90033, USA

**Keywords:** Sentinel lymph node biopsy, Breast cancer, Recurrence rates, DCIS, Ductal carcinoma in-situ, Palpable DCIS

## Abstract

**Background:**

Palpable ductal carcinoma in-situ (pDCIS) is a subset of DCIS presenting with a clinical mass. We hypothesized pDCIS would have more aggressive clinical and pathological features, and higher rates of recurrence and upgrade to invasive disease compared to screen-detected DCIS.

**Materials and methods:**

We performed a retrospective analysis of female patients (age 28–76) with DCIS on core-needle biopsy. pDCIS patients had a physician documented palpable mass prior to initial biopsy. Descriptive statistics were performed to compare groups.

**Results:**

This study included 83 patients, 26 had pDCIS and 57 had screen-detected DCIS. Mean duration of follow-up was 49.4 months. pDCIS patients had significantly larger lesions (*p* = 0.03) which were more frequently biopsied via ultrasound (*p* = 0.002). In multivariate analysis, pDCIS was associated with ultrasound guided core needle biopsy, size of DCIS >2 cm, and comedo pattern (*p* = 0.001, *p* = 0.007 and *p* = 0.022, respectively). 7.7 % of pDCIS cases versus 3.5 % of screen-detected cases were upgraded to invasive cancer (*p* = 0.59). There was no difference in local recurrence (*p* = 0.55) between groups. Neither group experienced regional or distant recurrence.

**Conclusions:**

pDCIS was associated with some aggressive pathologic and clinical features and was more frequently diagnosed by ultrasound guided core-needle biopsy than screen-detected DCIS. However, there was no significant difference in rate of recurrence or upgrade to invasive disease between groups.

**Key message:**

Although pDCIS was associated with some aggressive pathologic and clinical features, there was no significant difference in rate of recurrence or upgrade to invasive disease compared to screen-detected DCIS.

## Introduction

The incidence of ductal carcinoma in-situ (DCIS) has increased steadily since the introduction of screening mammograms and now accounts for approximately 25 % of new breast cancers [[1], [2], [3], [4]]. The majority of DCIS cases (>90 %) continue to be detected using imaging with a minority detected through symptoms such as a palpated mass [5]. Categorized as a “noninvasive cancer,” long-term survival of DCIS diagnosis following treatment is outstanding, exceeding 98 % after a follow-up of 10-years [6]. Amongst patients with DCIS who underwent treatment, recurrence rates vary from 5 to 20 %. However, about 50 % of such recurrences are found to be invasive and the risk of dying from breast cancer is three-folds greater for women diagnosed with DCIS compared to women within the same age group with no breast cancer diagnosis within a twenty year period [7,8].

Palpable DCIS (pDCIS) is a rare subtype of DCIS associated with a palpable mass; its incidence is uncertain. In contrast, screen-detected DCIS is often asymptomatic. Previous studies have shown pDCIS to be associated with more aggressive pathological features, greater risk of local treatment failure, and greater ipsilateral recurrence of invasive disease than screen-detected disease [9,10]. Many also believe pDCIS to infer a greater likelihood of upgrade to invasive disease on surgical pathology which may lead to additional surgical interventions and over treatment [11].

This retrospective chart review aimed to investigate if pDCIS cases demonstrated a more aggressive disease process than screen-detected DCIS cases at an urban safety-net institution, and to address potential implications of our findings on patient management.

## Materials and methods

### Database

This study was a retrospective chart review of patient visits to a single institution, urban safety-net medical center between January 1, 2006 and December 31, 2013. Cases were identified through the electronic medical record system at this medical center. Institutional approval was obtained, and no consent or waiver was required given the retrospective nature of this study.

### Methods

In this study, we included females aged 28–76 years who had pure DCIS without evidence of invasion on pathology on an initial core needle biopsy (CNB). Patients without a documented physical exam prior to undergoing CNB were excluded. We classified patient disease as pDCIS if a documented pre-CNB physical exam described a palpable lesion. Patients classified as screen-detected DCIS did not have a palpable lesion on the physical exam conducted prior to CNB. We then reviewed the patients' subsequent surgical pathology records to measure the upgrade rate to invasive disease. Patients with the following characteristics were excluded from this study: patients with micro-invasive or invasive disease found on CNB, patients with DCIS plus a concurrent invasive breast malignancy, and patients without available surgical pathology records*.*

### Patient characteristics

We recorded the following factors for each patient: 1) patient factors: age, race, ethnicity, personal history of breast cancer, family history of breast cancer, imaging and biopsy done with dates and results, and date of last follow-up; 2) tumor factors: tumor grade, estimated contiguous span of disease as recorded in the surgical pathology report, histologic type, presence of microcalcifications, location within the breast, size of surgical margin, receptor status (estrogen receptor (ER)/progesterone receptor (PR)), and local, regional, or distant recurrences; 3) treatment factors: lumpectomy, mastectomy, sentinel lymph node biopsy, use of radiation, and use of endocrine therapy.

### Statistical analysis

Statistical analyses were performed using SPSS software version 25.0 (SPSS, Inc., Chicago, IL, USA). Expectation-maximization method or frequent category imputation was used to handle missing values for several variables and multiple imputation was generated. The differences in clinicopathologic features of pDCIS compared to screen-detected DCIS were analyzed using the Chi-square test or the Fisher's exact test for categorical data and two sample *t*-test or the non-parametric Mann-Whitney *U* test for continuous data. Multivariate analysis was performed on significant variables at *p*-value <0.2 in a univariate analysis and those considered to be clinically relevant. Specifically, radiation therapy and endocrine therapy, although *p* = 0.13 and 0.16 respectively, were excluded from the analysis given the larger number of missing values. Additionally, variable inflation factors (VIF) for variables included in the multivariate analysis were not significant, so no adjustment was required regarding multicollinearity in the multivariate analysis. All tests were 2-sided and a *p*-value of <0.05 was considered to indicate a statistically significant difference.

## Results

A total of 83 cases were found to have DCIS on initial CNB; 26 cases (31.3 %) of these patients had a documented, palpable breast mass prior to CNB and were classified as pDCIS and 57 cases (68.7 %) had no documented palpable breast mass prior to CNB and were classified as screen-detected DCIS.

When comparing pDCIS patients to screen-detected patients in our study, there was no difference in patient race or ethnicity between groups. The majority of included patients with pDCIS and screen-detected DCIS identified as white in race (80.8 % vs 84.2 %) and Hispanic in ethnicity (69.2 % vs 80.7 %), reflective of the patient population typically treated at our institution. There was also no significant difference in personal or family history of breast cancer between groups. The majority of patients with pDCIS and screen-detected DCIS denied a previous personal history of breast cancer (92.3 % vs 91.2 %) and did not report a family history of breast cancer (84.6 % vs 73.7 %). Biopsy type differed significantly between pDCIS and screen-detected DCIS cases, as 13 (50 %) pDCIS patients underwent initial ultrasound-guided core needle biopsy whereas 47 patients with screen-detected DCIS (82.5 %) underwent initial stereotactic core needle biopsy (SCNB) (*p* = 0.002).

While we observed a variety of histologic profiles, most pDCIS and screen-detected DCIS patients demonstrated a similar solid papillary histologic pattern (69.2 % vs 61.4 %). Patients with pDCIS were, however, more likely to demonstrate a comedo pattern than those with screen-detected DCIS (30.8 % vs 15.8 %, *p* = 0.12). There was no difference in grade or hormone receptor status between groups with most patients having estrogen positive and or progesterone positive, and intermediate to high grade disease. Involvement of surgical margins was similarly rare across groups (0 % for pDCIS vs 8.8 % for screen-detected DCIS, *p* = 0.32), and there was also no significant difference in surgical mean margin size between groups (0.5 ± 0.6 cm vs 0.6 ± 0.5 cm, *p* = 0.5). However, pDCIS was associated with a larger mean size of DCIS, 2.7 ± 1.9 cm vs 1.7 ± 1.8 cm in the screen-detected group (*p* = 0.03).

From a treatment standpoint, the majority of patients in both groups underwent lumpectomy (69.2 % for pDCIS and 71.9 % for screen-detected DCIS), followed by radiation therapy (65.4 % for pDCIS and 80.7 % for screen-detected DCIS) (Table 1). Further analyzing the use of radiation therapy amongst all study patients, lumpectomy was associated with radiation therapy while mastectomy was not (*p* = 0.017 vs *p* = 0.117). However, there was no difference in radiotherapy by surgical method (lumpectomy versus mastectomy) when comparing patients with pDCIS to those with screen-detected DCIS (*p* = 0.158). Rate of sentinel lymph node biopsy was low for both groups, but higher amongst patients with palpable compared to screen-detected DCIS (42.3 % vs 29.8 %) (Table 1). Additionally, SLNB was further broken down by lumpectomy and mastectomy, with a significantly increased use of SLNB in patients undergoing mastectomy vs lumpectomy (68.2 % vs 21.3 %, *p* = 0.001) (Table 2). Further comparing SLNB in pDCIS vs screen-detected DCIS, there was a statistically significant difference in SLNB amongst the two groups in patients who underwent lumpectomy however not in those who underwent mastectomy (Table 3, Table 4). There was no significant difference in utilization of endocrine therapy between groups (96.2 % for pDCIS and 84.2 % for screen-detected DCIS) (Table 1).

**Table 1:** Characteristics of patients with palpable DCIS compared to non-palpable DCIS.

Variables	Values		
	Palpable DCIS	Screen-detected DCIS	*p*-value
Race, n (%)			0.32
White	21 (80.8)	48 (84.2)	
Black or African American	3 (11.5)	2 (3.5)	
Asian	2 (7.7)	7 (12.3)	
Ethnicity, n (%)			0.31
Hispanic	18 (69.2)	46 (80.7)	
Non-Hispanic	5 (19.2)	9 (15.8)	
African American	3 (11.5)	2 (3.5)	
Personal history of breast cancer, n (%)			0.87
Yes	2 (7.7)	5 (8.8)	
No	24 (92.3)	52 (91.2)	
Family history of breast cancer, n (%)			0.27
Yes	4 (15.4)	15 (26.3)	
No	22 (84.6)	42 (73.7)	
Biopsy type, n (%)			0.002⁎
Ultrasound guided core needle biopsy	13 (50.0)	10 (17.5)	
Stereotactic core needle biopsy	13 (50.0)	47 (82.5)	
Microhistology, n (%)			
Solid papillary	18 (69.2)	35 (61.4)	0.49
Comedo pattern	8 (30.8)	9 (15.8)	0.12
Cribriform	15 (57.7)	33 (57.9)	0.99
Micropapillary	5 (19.2)	5 (8.8)	0.27
Central Necrosis	7 (26.9)	17 (29.8)	0.79
Microcalcifications	6 (23.1)	16 (28.1)	0.63
Grade, n (%)			0.64
I (low)	4 (15.4)	14 (24.6)	
II (intermediate)	12 (46.2)	23 (40.4)	
III (high)	10 (38.5)	20 (35.1)	
Size of DCIS (mean), cm	2.7 ± 1.9	1.7 ± 1.8	0.03⁎
Size of DCIS			0.02⁎
≤ 2 cm	14 (53.8 %)	45 (78.9 %)	
>2 cm	12 (46.2)	12 (21.1 %)	
Involvement of surgical margins, n (%)			0.32
Yes	0 (0)	5 (8.8)	
No	26 (100)	52 (91.2)	
Surgical margin (mean), cm	0.5 ± 0.6	0.6 ± 0.5	0.5
Estrogen Receptor, n (%)			0.81
Positive	22 (84.6)	50 (87.7)	
Negative	4 (15.4)	7 (12.3)	
Progesterone Receptor, n (%)			0.98
Positive	20 (76.9)	44 (77.2)	
Negative	6 (23.1)	13 (22.8)	
Treatment strategy, n (%)			
Mastectomy	8 (30.8)	14 (24.6)	0.55
Lumpectomy	18 (69.2)	41 (71.9)	0.8
Radiation therapy	17 (65.4)	46 (80.7)	0.13
Endocrine therapy	25 (96.2)	48 (84.2)	0.16
Sentinel lymph node biopsy, n (%)	11 (42.3)	17 (29.8)	0.27
Postoperative diagnosis, n (%)			0.59
DCIS only	24 (92.3)	55 (96.5)	
Invasive cancer & DCIS	2 (7.7)	2 (3.5)	
Local recurrence, n (%)	0 (0)	3 (5.3)	0.55
Regional recurrence, n (%)	0 (0)	0 (0)	⁎⁎
Distant recurrence, n (%)	0 (0)	0 (0)	⁎⁎
Follow-up time (mean), months	46.6 ± 23.9	50.6 ± 20.4	0.43

⁎ Denotes statistical significance (*p* < 0.05).

⁎⁎ Denotes not applicable.

**Table 2:** SLNB in mastectomy vs lumpectomy.

Variables	Values		
	Mastectomy	Lumpectomy	*p*-value
SLNB, n (%)			<0.001⁎
SLNB	15 (68.2)	13 (21.3)	
No SLNB	7 (31.8)	48 (78.7)	

⁎ Denotes statistical significance (*p* < 0.05).

**Table 3:** SLNB in lumpectomy group in pDCIS vs screen-detected DCIS.

Variables	Values		
	Palpable DCIS	Screen-detected DCIS	*p*-value
SLNB in lumpectomy group, n (%)			0.043⁎
Lumpectomy + SLNB	7 (38.9)	6 (14.0)	
Lumpectomy + No SLNB	11 (61.1)	37 (86.0)	

⁎ Denotes statistical significance (*p* < 0.05).

**Table 4:** SLNB in mastectomy group in pDCIS vs screen-detected DCIS.

Variables	Values		
	Palpable DCIS	Screen-detected DCIS	*p*-value
SLNB in mastectomy group, n (%)			0.343
Mastectomy + SLNB	4 (50.0)	11 (78.6)	
Mastectomy + No SLNB	4 (50.0)	3 (21.4)	

We observed no difference in the upgrade rate from DCIS to invasive disease on final surgical pathology between pDCIS and screen-detected DCIS cases (7.7 % vs 3.5 % respectively, *p* = 0.59). There were 3 patients who experienced local recurrence, all of whom had screen-detected DCIS. However, there was no statistically significant difference in local recurrence between patients with pDCIS and screen-detected DCIS (*p* = 0.55), and no patients experienced regional or distant recurrence. There was no difference in mean follow-up time between groups (Table 1).

In a multivariate analysis, the adjusted odds ratio of statistically significant variables in pDCIS patients was 8.3 (95 % CI of 2.4–28.8) for ultrasound guided core needle biopsy, 5.36 (95 % CI of 1.59–18.07) for the size of DCIS >2 cm, and 4.83 (95 % CI of 1.25–18.65) for the presence of comedo pattern (*p =* 0.001, *p* = 0.007 and *p* = 0.022, respectively) (Table 5).

**Table 5:** Factors associated with palpable DCIS in the multivariate analysis (*n* = 48).

Variable	Palpable DCIS			
	Adjusted		Unadjusted	
	OR (95 % CI)	*p*-value	OR (95 % CI)	*p*-value
Biopsy type (ultrasound guided core needle biopsy)	8.30 (2.4–28.8)	0.001⁎	4.70 (1.68–13.14)	0.003⁎
Size of DCIS >2 cm	5.36 (1.59–18.07)	0.007⁎	3.21 (1.18–8.74)	0.022⁎
Comedo pattern	4.83 (1.25–18.65)	0.022⁎	2.37 (0.79–7.09)	0.123

⁎ Denotes statistical significance (*p* < 0.05).

## Discussion

Given its relative rarity, there is a paucity of literature regarding palpable DCIS. More commonly referenced in the literature is “symptomatic DCIS” with palpability being one of several symptomatic presentations. Furthermore, documentation as to whether the patient has a palpable breast mass prior to undergoing biopsy is frequently lacking. As a result, management of pDCIS is variable. Our study focused specifically on the symptom of palpability. We also required a physical examination of the patient prior to CNB providing a more accurate designation of palpable versus non-palpable disease. Our findings suggest that pDCIS does not represent a definitively more aggressive disease process than screen-detected DCIS.

Biopsy type between the two groups differed significantly, with 50 % of pDCIS patients biopsied using ultrasound guided core needle biopsy compared to 82.5 % of screen-detected DCIS using SCNB (*p* = 0.002). It is not surprising that ultrasound was preferentially used to guide biopsy for pDCIS cases given that pDCIS is more likely to have an ultrasonographically visible mass to target compared to screen-detected DCIS which is most frequently detected via calcifications. However, there is not consensus regarding detection of pDCIS with ultrasound versus mammography which helps to explain why pDCIS cases were not solely biopsied via ultrasound guidance. While ultrasound was found to be more sensitive than mammography in detecting mass lesions in pDCIS in some studies, pDCIS was more frequently visible on mammography than sonography in other studies [9,12]. Furthermore, although DCIS is routinely pre-operatively diagnosed by SCNB, this modality is known to sometimes underestimate the incidence of invasive disease since microcalcifications are most frequently targeted. For example, a paper by Lee et al. reported that of 59 patients diagnosed with DCIS by SCNB, 29 % were subsequently found to have invasive disease after surgery [13]. In our cohort of pDCIS patients, there was no significant difference in upgrade rate to invasive breast cancer compared to patients with screen-detected DCIS, and the rate of upgrade to invasive malignancy was rare. This suggests that in the contemporary era, percutaneous core biopsy histology may more accurately risk stratify pDCIS lesions.

Treatment strategies for DCIS are varied and include mastectomy, lumpectomy, radiotherapy, and endocrine therapy. Staging and treatment selection can be informed by risk factor profiles and evaluation of axillary lymph nodes via SLNB [14]. Such risk factors include presence of a palpable mass, surgical margins <2 mm, large span of disease on imaging (≥ 4 cm), high nuclear grade, presence of comedo necrosis or comedo pattern on histology, and lack of hormone receptor expression [5,9,[15], [16], [17], [18], [19]]. The presence of such risk factors increases the likelihood that patients will be recommended for mastectomy, SLNB, and/or adjuvant radiation therapy [11,16,17,[20], [21], [22], [23]]. In our study, surgical margins, nuclear grade, and receptor status were similar between pDCIS and screen-detected DCIS patients. However, patients with pDCIS did have a larger span of disease on final surgical pathology and were more likely to have a comedo pattern on microhistology compared to patients with screen-detected DCIS, suggesting potentially more aggressive disease. Of import, the average pDCIS cancer in our study was <4 cm and still amenable to lumpectomy. In our patient cohort there was no statistically significant difference in surgical treatment strategy for patients with pDCIS compared to those with screen-detected DCIS, with most patients undergoing lumpectomy. Additionally, while most patients with pDCIS and screen-detected DCIS did not undergo sentinel lymph node biopsy, SLNB was significantly more commonly employed amongst mastectomy compared to lumpectomy patients. Furthermore, SLNB was significantly more commonly performed at the time of lumpectomy in pDCIS patients compared to screen-detected DCIS. This was not the case when comparing SLNB in pDCIS patients to screen-detected DCIS patients undergoing mastectomy.

Many of the risk factors that increase the likelihood of a patient being recommended for SLNB and radiation therapy also portend an increased risk of breast cancer recurrence. A systematic review of the literature with meta-analysis performed by Visser et al. demonstrated that African American race, premenopausal status, detection by palpation, involved margins, and high histologic grade are all associated with invasive recurrence [24]. Endocrine therapy and radiation therapy are adjuvant treatment strategies utilized in the setting of DCIS to reduce the risk of breast cancer recurrence. In our study there was no statistically significant difference between adjuvant therapy in the pDCIS and screen-detected DCIS groups, with most patients treated with adjuvant radiation and endocrine therapy. The results of both our study and that done by Rajan et al. showed that the majority of pDCIS cases had ER-positive disease [9]. Therefore, the use of endocrine therapy would thus be particularly applicable and beneficial to the women in these studies and potentially to patients with pDCIS in general.

The upgrade rate to invasive cancer in the setting of pDCIS is variable in the literature. Invasive disease was noted at time of surgical pathology in 15 % (*n* = 6) of pDCIS cases by Rajan et al.*,* 54 % (*n* = 25) of pDCIS patients by Barnes et al., and 59 % (*n* = 19) of pDCIS patients by Yen et al. [9,16,18] These studies document an upgrade rate to invasive disease in the setting of pDCIS that is higher than the 7.7 % (n = 2) noted in our study. We found no statistically significant difference in upgrade rate to invasive disease between pDCIS and screen-detected DCIS. Palpability was also not a statistically significant predictor for upgrade to invasive breast cancer noted by Yen et al. nor Barnes et al., and this was not assessed by Rajan et al. [9,16,18]

Given that the majority of DCIS cases do not progress to invasive disease, overtreatment remains a clinical concern even when comparing the management of DCIS and pDCIS. In addition to the physical and financial adverse outcomes many patients have to endure from treatment, studies have shown that a DCIS diagnosis is associated with equal levels of anxiety and depression and similar reductions in physical and mental quality of life compared to low-grade invasive breast cancer, underscoring that there are behavioral and psychological factors to consider as well [25]. Therefore, there is a great opportunity to consider the many different predictors and various outcomes of DCIS and integrate such information to create a reliable guide for clinicians to combat overtreatment without compromising outcomes. While patients with pDCIS in our study had more aggressive pathology (comedo pattern) and larger cancers than those with screen-detected DCIS, patients in both groups also had several other clinical and pathologic features suggestive of similar clinical risk. Furthermore, rates of invasive disease found on surgical pathology and local, regional, and distant recurrence were similar amongst the two groups.

This discussion of overtreatment is important specifically in regards to SLNB given the trend in de-escalation of axillary surgery. Decisions for undergoing SLNB are multifactorial but have been influenced by presence of a palpable mass due to concern for higher upgrade rate to invasive disease and the desire to spare patients a second anesthetic event and surgery if lumpectomy is performed. In 2001, the Consensus Conference on the role of sentinel lymph node biopsy in carcinoma of the breast stated that SLNB may be indicated in patients whose DCIS presents as a palpable breast mass if planned treatment was that of mastectomy or large lumpectomy given the concern for finding invasive cancer on surgical pathology [11]. Per NCCN guidance, “the performance of a SLN procedure should be considered if the patient with apparent pure DCIS is to be treated with mastectomy,” given concern for impaired ability to accurately assess the axillary drainage pattern of the breast following mastectomy [26]. While the majority of our patients did not undergo SLNB, 42.3 % of pDCIS patients and 29.8 % of screen-detected DCIS patients did undergo this procedure. While high rates of SLNB in our patient cohort are influenced by the large span of disease and concomitant concern for sampling error, as well as the rate of mastectomy for surgical management, that pDCIS patients treated with lumpectomy more commonly underwent SLNB compared to screen-detected DCIS patients treated with lumpectomy (38.9 % vs 14.0 %, *p* = 0.043) suggests an anticipated higher rate to invasive disease expected amongst providers. Given the trend towards de-escalation of axillary surgery and the low upgrade rate to invasive cancer with pDCIS which we document in our paper, we think SLNB in patients with pDCIS is unlikely to add to clinical care and may lead to increased morbidity, cost, and difficulty in nodal evaluation if additional breast disease occurs in the future [17,26,27].

Our study includes a modest sample size. A contributing factor for the limited number of DCIS cases in this study compared to other analyses is the patient demographic served by our institution. Because medically underserved patients have decreased access to care, which can result in decreased adherence to health screenings, patients often present with more advanced, invasive disease rather than DCIS. Another factor likely contributing to the limited number of total DCIS cases in our study is the requirement of a documented physical examination prior to undergoing core needle biopsy. Thus, many DCIS cases seen at our institution were excluded as there was no documented exam prior to biopsy.

Given the small sample size, our results should be corroborated with a prospective registry for pDCIS. We particularly anticipate the Comparison of Operative versus Monitoring and Endocrine Therapy (COMET) trial results, an ongoing randomized trial aiming to evaluate the ipsilateral invasive breast cancer rate in low-risk DCIS patients who undergo guideline coordinate care vs active surveillance, thereby directly addressing the question of overtreatment in screen-detected DCIS [28]. However, the COMET trial excludes patients with a documented mass on examination and given its rarity, a randomized trial assessing treatment outcomes for pDCIS would not be realistic at this time.

## Conclusion

Cases of pDCIS did not demonstrate more aggressive disease than screen-detected DCIS based on our institutional experience. Additionally, there was no difference in upgrade rates to invasive disease on surgical pathology nor higher recurrence rates between the two groups. Our study did show that ultrasound guided core needle biopsy was used more frequently for pDCIS cases and SCNB was used more frequently for screen-detected DCIS cases. This suggests that in the contemporary era, percutaneous core needle biopsy histology may accurately risk stratify lesions and that palpability in Stage 0 breast cancer may not portend any worse prognosis. Given concern for overtreatment of DCIS and its implications, we encourage further research aimed at challenging the dogma that pDCIS has more adverse tumor biology than screen-detected DCIS.

## Abbreviations

BCS: breast conserving surgery
BCS + RT: breast conserving surgery with radiation therapy
DCIS: ductal carcinoma in situ
pDCIS: palpable ductal carcinoma in situ
ER: estrogen receptor
PR: progesterone receptor
SLNB: sentinel lymph node biopsy
CNB: core needle biopsy
SCNB: stereotactic core needle biopsy
COMET: Comparison of Operative versus Monitoring and Endocrine Therapy

## CRediT authorship contribution statement

Nina Balac and Robert M. Tungate contributed to the writing, data collection, and design of the project. Young Ju Jeong contributed to the statistical analysis. Heather MacDonald, Lily Tung, Naomi Schechter, Linda Larsen, and Stephen Sener contributed to critical revisions of the manuscript and study design. Kirstyn E. Brownson contributed to the writing, analysis, design, and critical revisions for the project. Julie Lang contributed to the writing, analysis, design and study conception.

## Funding

The project described was supported in part by award number P30CA014089 from the 10.13039/100000054National Cancer Institute.

## Ethics approval

This project was submitted for ethical review to the Institutional Review Board (IRB) at the University of Southern California and IRB approval was obtained.

## Declaration of competing interest

The authors report no proprietary or commercial interest in any product mentioned or concept discussed in this article.
